# Gain control via feedforward inhibition in noisy and delayed neural circuits

**DOI:** 10.1186/1471-2202-15-S1-P111

**Published:** 2014-07-21

**Authors:** Jorge F Mejias, Alexandre Payeur, Erik Selin, Leonard Maler, Andre Longin

**Affiliations:** 1 Center for Neural Science, New York University, New York, NY, 10012, USA; 2 Department of Physics, University of Ottawa, Ottawa, ON, K1N6N5, Canada; 3 Department of Cellular and Molecular Medicine, University of Ottawa, Ottawa, ON, K1H8M5, Canada; 4 Centre for Neural Dynamics, University of Ottawa, Ottawa, ON, K1N6N5, Canada

## 

The control and scaling of the input-output behavior of neural networks, or gain control, is one of the main strategies used by neural systems for the processing and gating of information. This input-output behavior is often described by the so-called f-I curve, which shows the output firing rate as a function of the input current to the neuron or neural circuit [1]. In particular, the slope of such a dependency constitutes a useful marker of the behavior of the neuron. If the slope of the f-I curve is high, small changes in the input current will be mapped by the cell into large changes in the output firing rate, which implies an increasing of the sensitivity of the neuron to weak stimuli. On the other hand, a small slope of the f-I curve translates large changes in the input current to small changes in the output firing rate, allowing the neuron to encode a broad range of stimulus intensities into a narrow range of firing rates.

Several gain control behaviors have been found to be particularly relevant for the gating and transformation of information in neural circuits. For instance, common biological mechanisms have been found to produce subtractive effects in the f-I curve [2], while a few others are thought to induce divisive (i.e. changes in the slope of the f-I curve)or other nonlinear effects [2-4]. However, a common mechanism able to induce these three broad classes of gain control has not been described up to date.

We present here (see [5] for further details) a study of gain control in a feedforward neural circuit inspired by the electrosensory lateral-line lobe of weakly electric fish. Our model displays three different gain control regimes: subtractive, divisive and non-monotonic. The neural circuit can shift from one regime to the other by a simple modulation of the synaptic strength of the inhibitory feedforward pathway present in the circuit, which, in the electrosensory circuit used as example, is known to present long-term synaptic plasticity mechanisms. To our knowledge, this is the first example of a network which presents all these gain controls at once. We further study the effect of noise and delays on this gain control mechanism, showing in particular that delays in the feedforward inhibitory pathway linearize the input-to-output relationship of the network, acting as an extra source of noise in this sense. Finally, using physiological evidences, we apply our model to the case of the divisive gain control observed *in vivo* in weakly electric fish.

Our work illustrates how subtractive, divisive and non-monotonic gain control may be obtained in inhibitory feedforward neural circuits. In addition, the analysis of the conditions in which the f-I response curve of superficial pyramidal neurons becomes non-monotonic reveals a novel nonlinear gain control mechanism which agrees with *in vitro* experimental recordings in the electric fish [6].
